# Clinical features and outcomes of non-pulmonary unifocal adult Langerhans cell histiocytosis

**DOI:** 10.1038/s41408-022-00685-7

**Published:** 2022-06-03

**Authors:** Marie Hu, Gaurav Goyal, Jithma P. Abeykoon, Aldo A. Acosta-Medina, Gordan J. Ruan, Jason R. Young, Aishwarya Ravindran, N. Nora Bennani, Mithun V. Shah, Robert Vassallo, Jay H. Ryu, Caroline J. Davidge-Pitts, Matthew J. Koster, W. Oliver Tobin, Julio C. Sartori-Valinotti, Karen L. Rech, Ronald S. Go

**Affiliations:** 1 Department of Internal Medicine, Mayo Clinic, Rochester, MN USA; 2 Division of Hematology, Mayo Clinic, Rochester, MN USA; 3 Department of Radiology, Mayo Clinic, Rochester, MN USA; 4 Department of Laboratory Medicine and Pathology, Mayo Clinic, Rochester, MN USA; 5 Division of Pulmonary and Critical Care Medicine, Mayo Clinic, Rochester, MN USA; 6 Division of Endocrinology, Diabetes, and Nutrition, Mayo Clinic, Rochester, MN USA; 7 Division of Rheumatology, Mayo Clinic, Rochester, MN USA; 8 Department of Neurology, Mayo Clinic, Rochester, MN USA; 9 Department of Dermatology, Mayo Clinic, Rochester, MN USA; 10 Present Address: Division of Hematology, Oncology, and Transplantation, University of Minnesota, Minneapolis, MN USA; 11 Present Address: Division of Hematology-Oncology, University of Alabama at Birmingham, Birmingham, AL USA

**Keywords:** Haematological cancer, Disease-free survival, Haematological diseases

Dear Editor,

Langerhans cell histiocytosis (LCH) is a rare hematologic disorder affecting adults with an estimated incidence of 1–2 cases per million [1]. The clinical presentation varies widely, with the most common organs involved being bone, skin, and pituitary gland [2, 3]. After discovery of *BRAF V600E* and *MAP2K1* gain-of-function mutations in >70% of LCH patients, LCH was reclassified as an inflammatory myeloid neoplasm in the 2016 Histocyte Society classification [4–6]. In this revised classification, LCH was divided into single-system, pulmonary, or multisystem with or without risk organ involvement [4]. Unifocal (single lesion in one organ) LCH has previously been described as “self-limited”, but has not been well-studied in adults to date [7]. The existing knowledge in unifocal LCH is largely derived from historical case series or limited organ-specific case series [8–10]. In this study, we describe the clinical features and contemporary outcomes of adult patients with unifocal LCH from a single institution.

The study proposal was reviewed and approved by the Institutional Review Board. We retrospectively reviewed the medical records of 189 adult patients (≥18 years old at diagnosis) with histopathologically confirmed LCH who were seen at our institution between 1997 and 2018. Of these, we identified 44 patients who met the criteria for unifocal LCH at diagnosis (Fig. S1). To exclude other potential sites of disease at diagnosis, patients had undergone a thorough history and physical, complete blood count, serum chemistries, and at minimum dedicated lung imaging with chest X-ray (CXR) or computed tomography (CT) in accordance with the existing LCH guidelines for adults [3]. “High-risk” organs were defined as liver, spleen, bone marrow, or central nervous system (CNS) involvement [3]. Immunohistochemical staining for *BRAF V600E* or whole-exome sequencing (Illumina HiSeq4000; San Diego, CA) was performed on any available patient specimens. Treatment response was assessed in terms of overall response rate (ORR), which encompasses partial response (PR) and complete response (CR) as previously defined [11]. For this study, we captured the best response, either clinically or radiographically. Overall survival (OS) and progression-free survival (PFS) were estimated using the Kaplan–Meier method with 95% confidence intervals (CI) and compared using the log-rank test.

We included 44 patients with unifocal LCH in the study (Tables 1 and S1). Median age at diagnosis was 42 years and 84% of patients were Caucasian. Fifty percent of patients were former or current smokers, with a median 18 pack-year history. The most common presenting symptoms were headache or skull pain/swelling (25%), rash/skin lesion (23%), abdominal pain or diarrhea (9%), and polyuria/polydipsia suggestive of diabetes insipidus (9%). The sites most commonly involved included bone (43%), skin (25%), hypothalamic-pituitary axis (HPA, 14%), and gastrointestinal tract (12%) (Figs. S2–4). Other rarer sites included lymph node, conjunctiva, and cervix. The median time from symptom onset to diagnosis was 2.6 months for the entire cohort and much longer at 28.6 months for HPA disease. In total, 18 patients in our cohort were tested for a *BRAF V600E* mutation, with 8 positive, 8 negative, and 2 equivocal. Additional information regarding organ involvement and BRAF testing are available in the Supplemental Appendix.

**Table 1:** Organ involvement and presenting symptoms at diagnosis in unifocal adult Langerhans cell histiocytosis.

Systems involved	# of cases (*n* = 44)	Specific sites	Presenting symptoms
Bone	19 (43%)	Cranium (11)	Skull pain/swelling (9)
		Vertebrae (3)	Back pain (3)
		Mandible (1)	Joint pain (2)
		Orbit (1)	Decreased hearing (2)
		Femur (1)	Rib pain (1)
		Rib (1)	Eye swelling (1)
		Pelvis (1)	Periodontal disease (1)
Skin	11 (25%)	Face (2)	Skin lesion NOS (4)
		Upper extremity (2)	Non-pruritic papule (1)
		Lower extremity (2)	Erythematous papules (1)
		Vulva (2)	Pedunculated papule (1)
		Chest (1)	Acneiform papule (1)
		Flank (1)	Hyperpigmented linear papule (1)
		Scalp (1)	Non-healing papule with central ulceration (1)
			Pruritic lesion NOS (1)
Hypothalamic-pituitary axis	6 (14%)	Hypothalamus (3)	Polyuria/polydipsia (4)
		Pituitary (2)	Headache (2)
		Infundibulum (1)	Weight gain (2)
			Menstrual changes (1)
			Cognitive changes (1)
Gastrointestinal	5 (12%)	Colon (3)	Abdominal pain (2)
		Rectum (1)	Diarrhea (2)
		Stomach (1)	Melena (1)
			Asymptomatic (1)
Lymph node	1 (2%)	Inguinal (1)	Groin swelling (1)
Reproductive	1 (2%)	Cervix (1)	Cervical nodule (1)
Mucosa	1 (2%)	Conjunctiva (1)	Eye itching (1)

Of the 44 unifocal adult LCH patients in our cohort, 5 patients were lost to follow-up and 1 patient did not receive LCH-directed therapy due to concurrent Hodgkin lymphoma. Therefore, treatment and follow-up data were available for 38 patients (Fig. 1; Tables S2 and S3). The most common first-line therapies were resection in 24 patients (63%) with ORR of 100% and focal radiation in 6 patients (16%) with ORR of 83%. Other less utilized first-line treatments included resection with adjuvant radiation, topical therapies for localized skin disease, high-dose dexamethasone, and cladribine. Smoking cessation/observation in 1 patient with a vertebral bone lesion resulted in a CR with no recurrence.

**Fig. 1:**
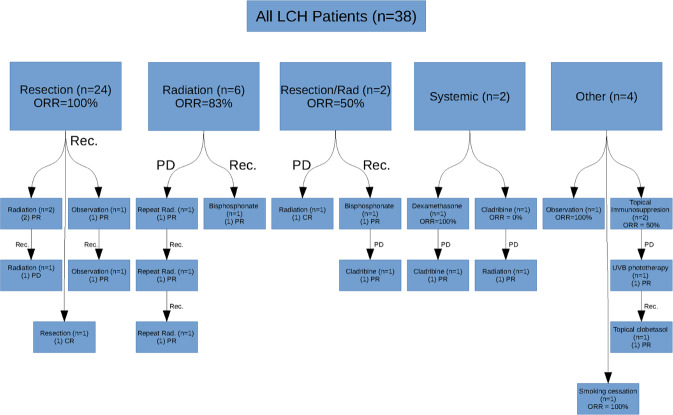
Flowchart of treatment and outcomes of unifocal adult Langerhans cell histiocytosis. ORR overall response rate, PD progressive disease, rec recurrence, PR partial remission, CR complete remission, rad radiation, UVB ultraviolet B.

Three patients (8%) had refractory/progressive disease and 10 (26%) developed recurrent disease after initial response to first-line therapy. Of the recurrences, 6 (55%) occurred at a new site within the same organ system and 5 (45%) occurred in a different organ system. The median time from start of first treatment to recurrence was 1.5 years. Patients with initially unifocal bone or skin LCH accounted for 80% of the recurrences with a recurrence rate of 25% for bone disease and 36% for skin disease. Four patients required third-line treatment, and only one patient underwent fourth-line treatment. The most common second/subsequent line therapy was localized radiation in 8 cases (largely for resistant or new bone lesions) with an ORR of 75%. Cladribine was used in one patient with subsequent multisystem disease and in one patient with hypothalamic disease that progressed on dexamethasone; both patients achieved PR with no further progression of disease at last follow up. Other later line treatments included bisphosphonates, ultraviolet B phototherapy, and observation.

The median duration of follow-up for the entire cohort was 7.3 years. Five patients were lost to follow-up immediately after their initial consultation at our institution, at a median of 1.6 months from diagnosis. The remaining 39 patients were all followed clinically, of whom 38% also underwent surveillance imaging within 1 year of last follow up visit (18% CT, 13% magnetic resonance imaging, and 7% positron emission tomography (PET)-CT). At last follow-up, 28 patients remained classified as unifocal, 6 patients were reclassified as single-system multifocal (4 bone, 2 skin), and 5 patients were reclassified as multisystem disease (Figs. S5 and S6). Median PFS after first-line therapy was not reached and 5-year PFS after first-line therapy was 68% (95% CI 50–81%) (Fig. S7). There was no difference in PFS after first-line therapy (*p* = 0.57) between patients who were *BRAF V600E* positive versus negative. Four patients died, with only one death from progressive LCH involving lymph nodes; other causes of death included refractory Hodgkin lymphoma, stroke, and unknown causes. The median OS for our cohort was not reached and 5-year OS was 94% (95% CI 79–99%) (Fig. S7).

This is the largest contemporary study of non-pulmonary unifocal adult LCH. Similar to pediatric disease, the most common organ systems involved were bone, skin, and hypothalamic-pituitary axis. The most common first-line treatment modalities consisted of either local resection or radiation, which were very effective in achieving a CR or PR; only one patient had a mandibular bone lesion that did not respond to radiation therapy. Only one patient treated with resection or radiation developed local recurrence, but about one in five patients developed disease recurrence at a new site. Also of interest is the observation that one patient with vertebral LCH experienced CR following smoking cessation, although it is possible that he experienced spontaneous healing as well. Systemic therapies were rarely utilized as first-line; both patients treated with either dexamethasone or cladribine as first-line treatment for pituitary disease had refractory/progressive disease. However, cladribine as second-line therapy in two cases (including one who developed multisystem disease) resulted in PR with no further recurrence.

Despite a small subset of patients that developed recurrent disease in our cohort, no patients developed pulmonary LCH or “high-risk” organ involvement (liver, spleen, bone marrow, or CNS). This suggests that unifocal, low-risk multisystem, high-risk multisystem, and pulmonary LCH may be biologically distinct entities. LCH has now been found to harbor MAPK-ERK pathway mutations in a majority of cases, with *BRAF V600E* mutations in 50–60% and *MAP2K1* mutations in 10–20% [5, 6]. In our study, only one *BRAF V600E* positive patient out of all patients tested for *BRAF* developed multisystem (low-risk) disease, with no association between the mutation status and PFS. Recently the “misguided myeloid differentiation model” of LCH ontogeny has been proposed, which hypothesizes that the differentiation level of the dendritic cell (DC) precursor that acquires an activating MAPK-ERK pathway mutation determines the extent and aggressiveness of subsequent disease [12, 13]. Our findings may support this hypothesis that low-risk single-lesion disease arises from mutated lesional tissue-restricted precursor DCs rather than blood or bone marrow progenitor DCs, and therefore carries a low risk of progression to high-risk multisystem disease.

In the largest adult LCH series put together by the International Histiocyte Society Registry in 2003, the subset of patients with single-system disease had a 5-year OS of 100% [2]. Otherwise, much of the current knowledge regarding unifocal LCH comes from pediatric literature, where the unifocal disease course has been described as benign. OS also approaches 100% and disease-free survival after first therapy is around 80–90% for unifocal/single-system LCH in most pediatric series [14, 15]. Outcomes in our adult cohort were slightly worse but overall comparable. However, in contrast to pediatric series where most recurrences occurred within the same organ system, the recurrences in our adult cohort were nearly evenly split between the same system versus another system (multisystem disease). This should be kept in mind when monitoring for recurrent disease.

Although there are no standard surveillance recommendations for adult unifocal LCH specifically, the 2013 Euro-Histio-Net guidelines for single-system LCH recommend monitoring history and labs every 6 months for 2 years followed by annually for 3 years and chest X-ray annually for 3 years [3]. In our cohort, only one patient with hypothalamic LCH developed late recurrence 6.6 years out with gastrointestinal and gum lesions. Therefore, we agree with the Euro-Histio-Net guidelines for close monitoring for at least the first 5 years, with potential to space out follow-up visits thereafter. In the absence of prospective surveillance studies, follow-up visits should focus on elucidating any symptoms that suggest recurrence, with imaging as clinically indicated.

Our study has some notable limitations, which can be ascribed to it being of retrospective nature. The patients diagnosed before 2012 did not undergo staging studies by PET-CT scans. Molecular data on *BRAF V600E* was only available for slightly less than half of patients, which made it difficult to fully assess the role of BRAF status in outcomes.

In summary, our study shows that the prognosis in non-pulmonary unifocal adult LCH is very good with 5-year OS of 94%. Of the four deaths, only one was from progressive LCH and occurred in the pre-targeted therapy era. Our findings suggest that unifocal LCH may be a unique different biologic entity that has good prognosis in a large subset of patients. Future studies to delineate the molecular underpinnings that predict an aggressive disease phenotype are needed and are underway.

## Supplementary information

Supplemental Appendix

## Data Availability

Data available on request due to privacy/ethical restrictions.
