# Supplementary material for: Clinical features and outcomes of non-pulmonary unifocal adult Langerhans cell histiocytosis
Source: Blood Cancer J. 2022 Jun 3;12(6):89. doi: 10.1038/s41408-022-00685-7 (PMC9166746; doi:10.1038/s41408-022-00685-7)
Supplement: Supplementary file 1 — Supplemental Appendix [file 41408_2022_685_MOESM1_ESM.docx]

**Supplementary Appendix**

Supplement to: Hu M, Goyal G, Abeykoon A, et al. “Clinical features and outcomes of non-pulmonary unifocal adult Langerhans cell histiocytosis.”

This appendix has been provided by the authors to give readers additional information about the work.

Organ involvement (detailed version)

In our cohort, the sites most commonly involved by unifocal LCH were bone (n=19, 43%), skin (n=11, 25%), hypothalamic-pituitary axis (n=6, 14%), and gastrointestinal tract (n=5, 12%). Other rarer sites included lymph node, conjunctiva, and cervix. (Figure S2)

- 1. *Bone*

Isolated bone disease was the most common manifestation of unifocal LCH in our cohort (43%). Interestingly, there was a male predominance with 13 male and 6 female patients. The specific sites involved were diverse but included the cranium most frequently (n=11; 5 parietal, 4 frontal, 1 temporal, and 1 occipital), followed by vertebrae (n=3; all thoracic spine), mandible (n=1), orbit (n=1), femur (n=1), ribs (n=1), and pelvis (n=1). Accordingly, the most common presenting symptoms were localized skull pain/swelling (n=9), back pain (n=3), and joint pains (n=2). For patients with cranial lesions, 45% (n=5) also had extension of the bony mass into the overlying soft tissue and 27% (n=3) had dural involvement. All patients underwent dedicated MRI or CT imaging (with characteristic punched-out lytic appearance) followed by biopsy for diagnosis, with 11 (58%) undergoing PET-CT or bone scan as well to rule out other bony lesions (Figure S3A, B, and D).

- 1. *Skin*

The next most common site of involvement was the skin (25%). There was a slight female predominance with 7 female patients and 4 male patients. Skin lesions were found in various locations, including face (n=2), upper extremity (n=2), lower extremity (n=2), vulva (n=2), chest (n=1), abdomen (n=1), and scalp (n=1). The skin lesions were largely described as single papules (1 small non-pruritic, 1 pedunculated, 1 acneiform, 1 hyperpigmented and linear, 1 non-healing with central ulceration) (Figure S3G); only one patient had multiple erythematous papules, covering a 1 cm area of the vulva. Five patients had skin lesions not otherwise specified (NOS) and two had associated pruritis. These were all detected clinically and confirmed by biopsy (punch or excisional).

- 1. *Hypothalamus and pituitary gland*

Six patients (14%) presented with isolated disease of the hypothalamic-pituitary axis, with sites of involvement including hypothalamus (n=3), pituitary gland (n=2), and infundibulum only (n=1). The sex distribution was equal between males and females. The median time from symptom onset to diagnosis was 28.6 months (IQR: 9.2-45.2), much longer than the entire cohort median of 2.6 months. The most common presenting symptoms were polyuria/polydipsia (n=4), headache (n=2), weight gain (n=2), menstrual irregularities (n=1), and cognitive changes (n=1). All patients were ultimately found to have diabetes insipidus, with additional evidence of anterior hypopituitarism in 3 patients and hypogonadism in 2 patients. All patients had visible lesions on brain MRI and underwent pituitary stalk/hypothalamus biopsies for diagnosis (Figure S3E and F, Figure S4). They were all treated by an endocrinologist for hormone therapy.

- 1. *Gastrointestinal tract*

Isolated gastrointestinal (GI) involvement was present in 5 patients (12%). Specific sites included the colon (n=3; 2 descending colon and 1 cecum), rectum (n=1), and gastric antrum (n=1). The most common presenting symptoms were abdominal/flank pain (n=2), diarrhea (n=2), and melena (n=1), which were most likely unrelated to LCH but led to the diagnostic work-up. One patient was asymptomatic but had a positive screening stool occult blood test which led to his colonoscopy. All patients underwent PET-CT or abdominal CT with contrast for further evaluation (Figure S3C). On endoscopy/colonoscopy, lesions were described as a single polyp or nodule which was fully resected and identified as LCH on pathology review after resection.

- 1. *Other sites*

There were 3 patients with unifocal LCH lesions of rarer sites, including conjunctiva (presented with eye itching), cervix (presented with a cervical nodule), and inguinal lymph node (presented with a groin lump/swelling).

*BRAF* testing (detailed version)

Initially, 5 patients in our cohort had been tested for a *BRAF V600E* mutation at diagnosis. These patients were all diagnosed with LCH during or after 2014. Therefore, additional immunohistochemical (IHC) staining for *BRAF V600E* or whole-exome sequencing (WES) was performed on any patient specimens that were available and had not been previously tested, leading to a total of 18 patients tested (8 had IHC only, 5 had WES only, 4 had both IHC and WES, and 1 had both IHC and tissue polymerase chain reaction). For IHC, the presence of homogeneous, granular, and strong cytoplasmic staining in the lesional cells was considered as ‘positive,’ while the presence of weak, heterogeneous, cytoplasmic and nuclear staining was considered as ‘equivocal.’ All IHC was reviewed by two hematopathologists (K.L.R. and A.R.). For whole-exome sequencing, sequencing was performed with paired-end sequencing (Illumina HiSeq4000; San Diego, CA) after target enrichment (SureSelect^XT HS^, Agilent; Santa Clara, CA).

In total, 18 patients in our cohort were tested for a *BRAF V600E* mutation. Eight patients were *BRAF V600E* positive (3 by IHC, 2 by WES, 2 by both IHC and WES, 1 by both IHC and tissue PCR); sites involved included bone (n=6), skin (n=1), and conjunctiva (n=1). Eight patients were *BRAF V600E* negative (4 by IHC, 3 by WES, 1 by both IHC and WES); sites involved included bone (n=5), hypothalamus (n=2) and GI (n=1). Two patients had equivocal results by IHC (1 bone, 1 hypothalamus), one of which was found to have a *BRAF* p.Arg662Lys mutation of unknown significance on WES.

**Table S1:** Baseline characteristics of patients with unifocal adult Langerhans cell histiocytosis.

| Characteristic | Value  (n=44) |
| --- | --- |
| Median age at diagnosis (yrs) | 42 (interquartile range: 34-52) |
| Sex |  |
| Female | 20 (45%) |
| Male | 24 (55%) |
| Race |  |
| White | 37 (84%) |
| Black | 0 (0%) |
| Asian | 0 (0%) |
| Other | 2 (5%) |
| Unknown | 5 (11%) |
| Smoking history |  |
| Yes | 22 (50%) |
| No | 16 (36%) |
| Unknown | 6 (14%) |
| BRAF status |  |
| V600E | 8 (18%) |
| No BRAF mutation | 8 (18%) |
| Other BRAF mutation | 1 (2%) |
| Equivocal | 1 (2%) |
| Unknown/not tested | 26 (59%) |
| Imaging at Diagnosis |  |
| PET or whole-body CT | 26 (60%) |
| MRI brain | 21 (48%) |
| Bone scan/skeletal survey | 25 (58%) |
| None of the above | 2 (5%) |

**Table S2:** Overall response rates and recurrence rates following first line treatment (by system) for unifocal adult Langerhans cell histiocytosis. ORR = overall response rate. SS = same system. DS = different system.

| Sites | Treatment (1st line)  (n=38) | ORR % | Recurrence  (local) | Recurrence  (new site SS) | Recurrence  (new site DS) |
| --- | --- | --- | --- | --- | --- |
| Bone | **Total (16)**  Resection (10)  Radiation (4)  Resection + radiation (1)  Smoking cessation (1) | **87% (14)**  100% (10)  75% (3)  0% (0)  100% (1) | **0% (0)**  0% (0)  0% (0)  0% (0)  0% (0) | **19% (3)**  20% (2)  0 (0)  100% (1)  0% (0) | **6% (1)**  0% (0)  25% (1)  0% (0)  0% (0) |
| Skin | **Total (11)**  Resection (8)  Resection + radiation (1)  Topical clobetasol (1)  Topical tacrolimus (1) | **91% (10)**  100% (8)  100% (1)  0% (0)  100% (1) | **9% (1)**  13% (1)  0% (0)  0% (0)  0% (0) | **18% (2)**  13% (1)  0% (0)  100% (1)  0% (0) | **9% (1)**  0% (0)  100% (1)  0% (0)  0% (0) |
| Hypothalamic-  Pituitary Axis | **Total (6)**  Resection (2)  Radiation (2)  Cladribine (1)  Dexamethasone (1) | **67% (4)**  100% (2)  100% (2)  0% (0)  0% (0) | **0% (0)**  0% (0)  0% (0)  0% (0)  0% (0) | **0% (0)**  0% (0)  0% (0)  0% (0)  0% (0) | **17% (1)**  100% (1)  50% (1)  0% (0)  0% (0) |
| Gastrointestinal | **Total (4)**  Resection (3)  Observation (1) | **100% (4)**  100% (3)  100% (1) | **0 (0)**  0% (0)  0% (0) | **0% (0)**  0% (0)  0% (0) | **0% (0)**  0% (0)  0% (0) |
| Mucosa | **Total (1)**  Resection (1) | **100% (1)**  100% (1) | **0% (0)**  0% (0) | **0% (0)**  0% (0) | **0% (0)**  0% (0) |

**Table S3:** Overall response rates and recurrence rates by treatment for unifocal adult Langerhans cell histiocytosis. ORR = overall response rate. Rec = recurrence. SS = same system. DS = different system. UVB = ultraviolet B.

| Treatment | 1st line | ORR% | Rec.  (local) | Rec.  (new site SS) | Rec.  (new site DS) | 2nd/later line | ORR % | Rec.  (local) | Rec.  (new site SS) | | Rec. (new site DS) |
| --- | --- | --- | --- | --- | --- | --- | --- | --- | --- | --- | --- |
| Resection | 24 | **100%** | 4% | 13% | 4% | 1 | **100%** | 0% | 0% | 0% | |
| Radiation | 6 | **83%** | 0% | 0% | 33% | 8 | **75%** | 13% | 13% | 13% | |
| Resection + radiation | 2 | **50%** | 0% | 50% | 50% | - |  |  |  |  | |
| Topical immunosuppression | 2 | **50%** | 0% | 50% | 0% | 1 | **100%** | 0% | 0% | 0% | |
| Systemic steroid | 1 | **0%** | - | 0% | 0% | - |  |  |  |  | |
| Cladribine | 1 | **0%** | - | 0% | 0% | 2 | **100%** | 0% | 0% | 0% | |
| Smoking cessation | 1 | **100%** | 0% | 0% | 0% | - |  |  |  |  | |
| Observation | 1 | **100%** | 0% | 0% | 0% | 2 | **50%** | 0% | 0% | 50% | |
| Bisphosphonates | 0 |  |  |  |  | 2 | **100%** | 0% | 0% | 50% | |
| UVB phototherapy | 0 |  |  |  |  | 1 | **100%** | 0% | 100% | 0% | |

**Figure S1:** Flow-chart of patient selection from all adult LCH patients to unifocal patients only.

**Figure S2:** Sites of involvement at diagnosis in unifocal adult Langerhans cell histiocytosis.


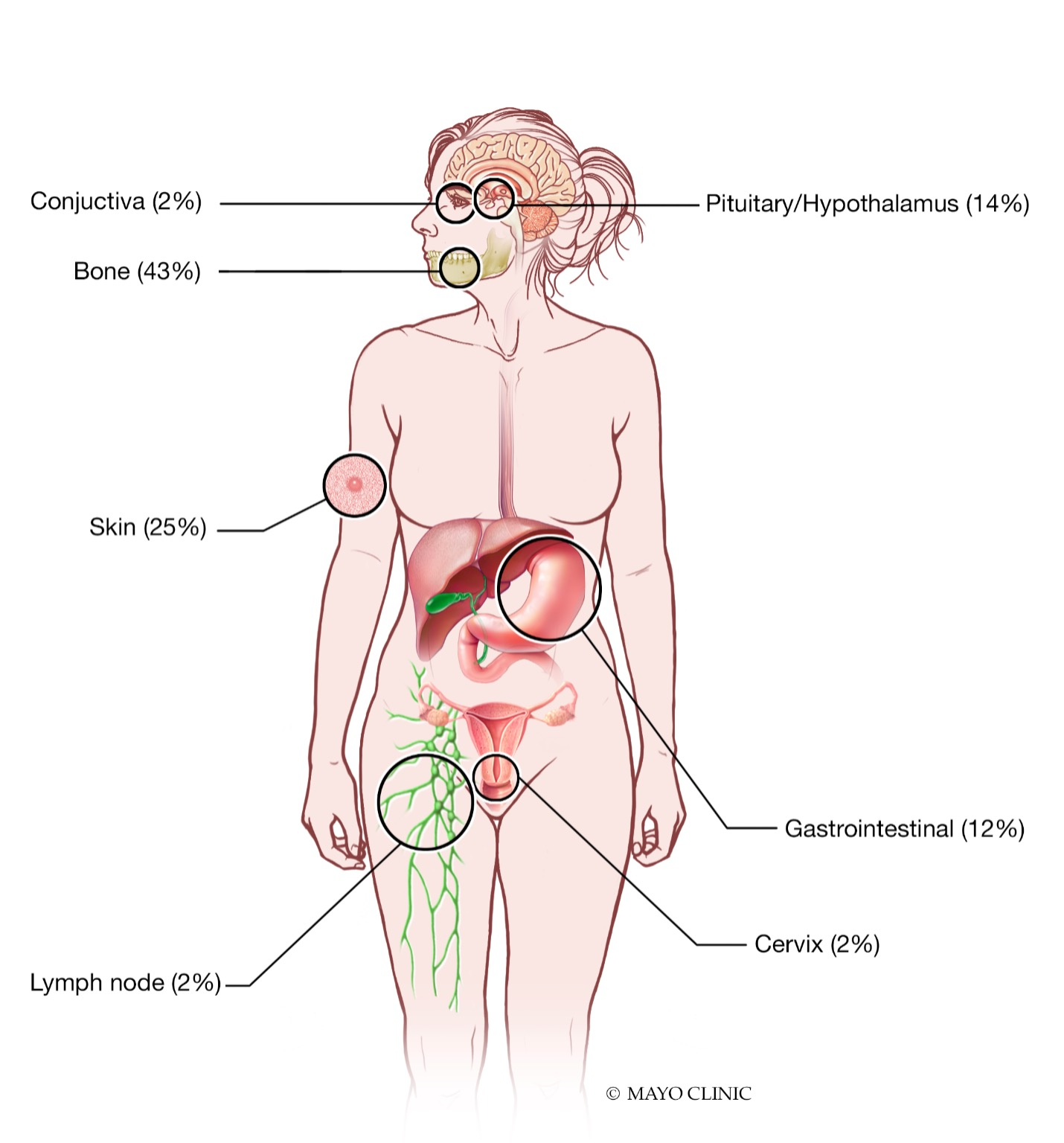


**Figure S3:** Different patients as examples of non-pulmonary unifocal Langerhans cell histiocytosis involvement. A, B) Axial fused FDG PET-CT of the skull demonstrating a large FDG avid (9.5 SUVmax) lytic lesion (arrow). C) Axial CT of the abdomen with enteric contrast outlining a polypoid mass projecting into the lumen of cecum (arrow). D) Coronal CT of the paranasal sinus showing a “floating tooth” sign of a lytic right maxillary sinus lesion (bracket). E) Coronal T1 weighted MRI of the pituitary with gadolinium contrast demonstrating mild enhancement of the posterior pituitary/infundibulum (arrow) and then 3 months later (F) an enhancing mass developed in the same region (arrow). G) Photograph of the lower leg highlighting a small pinkish papule that had a history of becoming crusted.


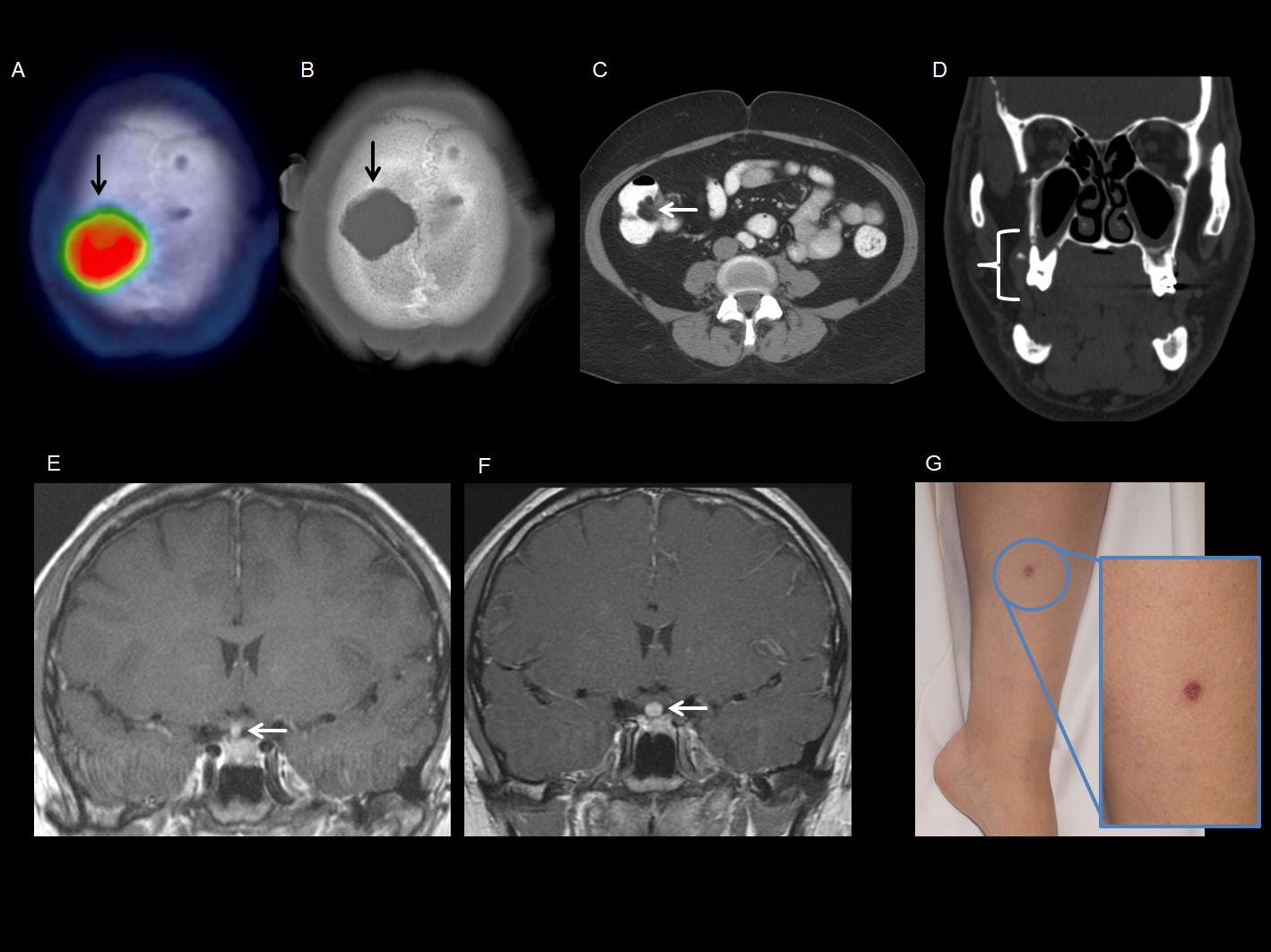


**Figure S4:** Biopsy (H&E stain) from a hypothalamic mass in a 30-year-old man presenting with headache, weight gain and decreased libido shows mixed inflammation including clusters of Langerhans cells, small lymphocytes and rare eosinophils (A: Hematoxylin and eosin). The Langerhans cells are positive for CD68 (B), CD1a (C) and Langerin (D) by immunohistochemistry. [All images taken at 200x magnification with Olympus Optical BX50F4 microscope and Olympus DP73 camera].


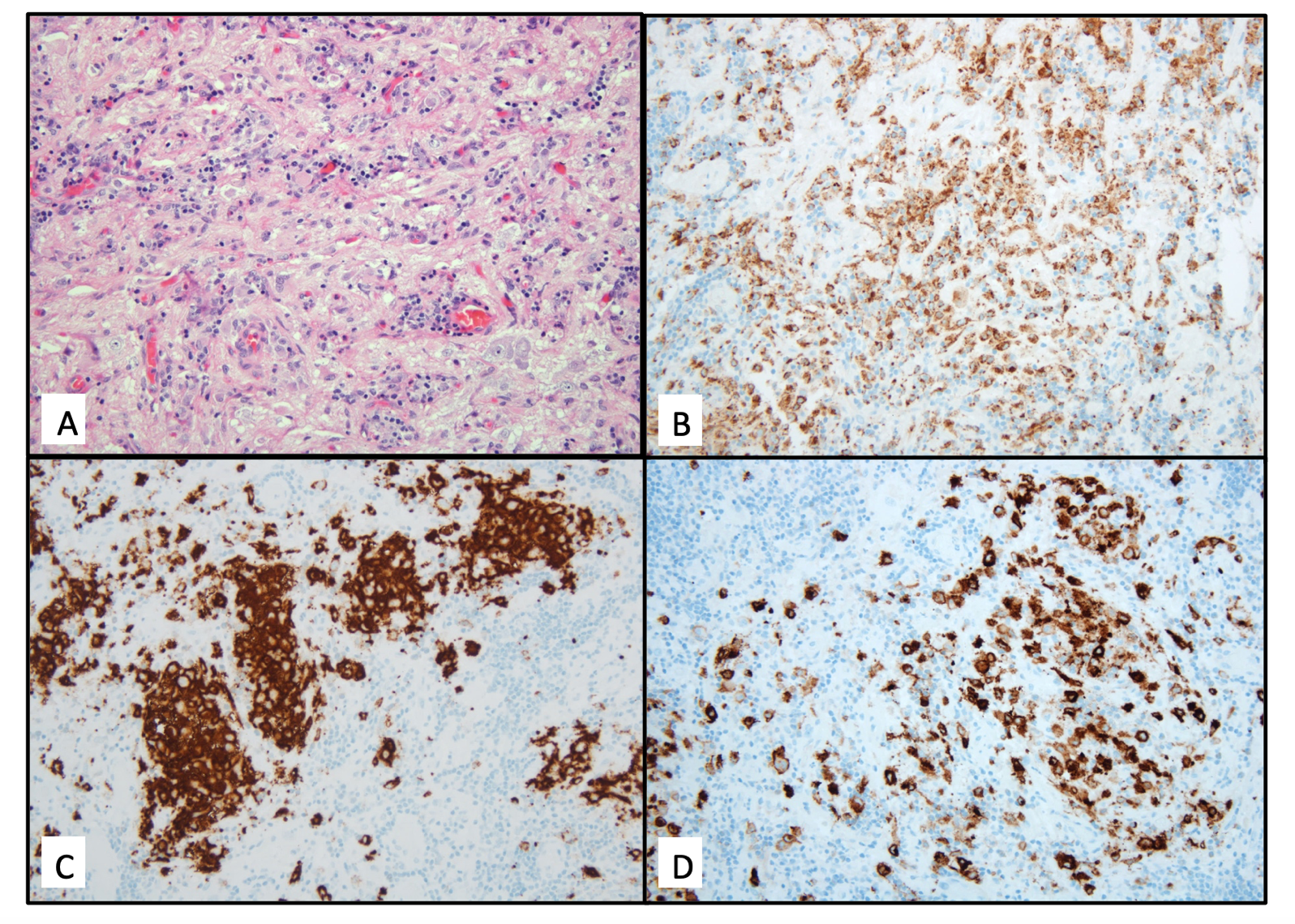


**Figure S5:** Evolution of unifocal adult Langerhans cell histiocytosis from time of diagnosis to time of last-follow-up (median follow-up 7.3 years)**.** GI = gastrointestinal. HPA = hypothalamic-pituitary axis. LN = lymph node.


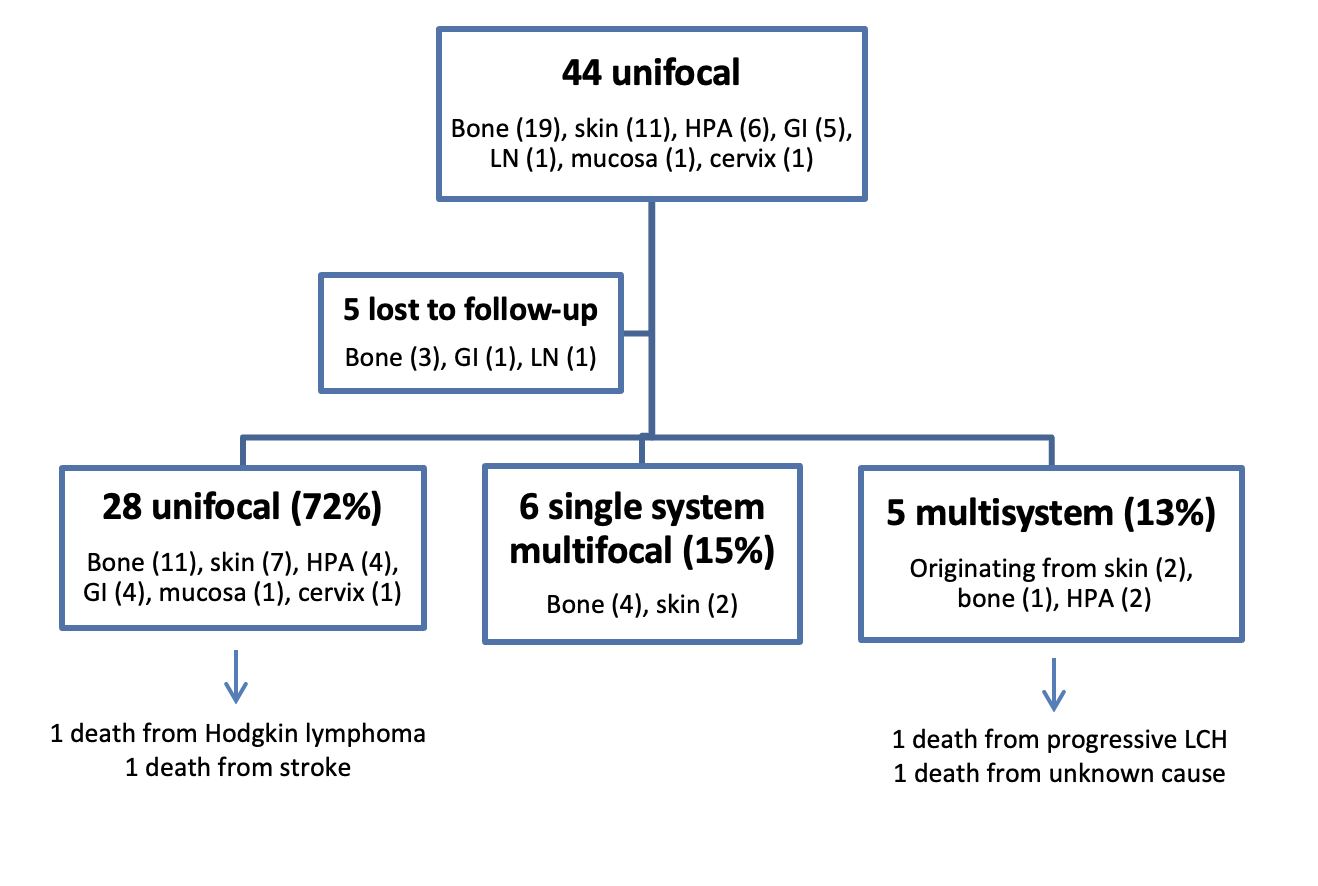


**Figure S6:** Evolution of unifocal adult Langerhans cell histiocytosis by system from time of diagnosis to time of last-follow-up (median follow-up 7.3 years)**.** GI = gastrointestinal. HPA = hypothalamic-pituitary axis. LN = lymph node. SSM = single-system multifocal. MS = multisystem. F/u = follow-up. **
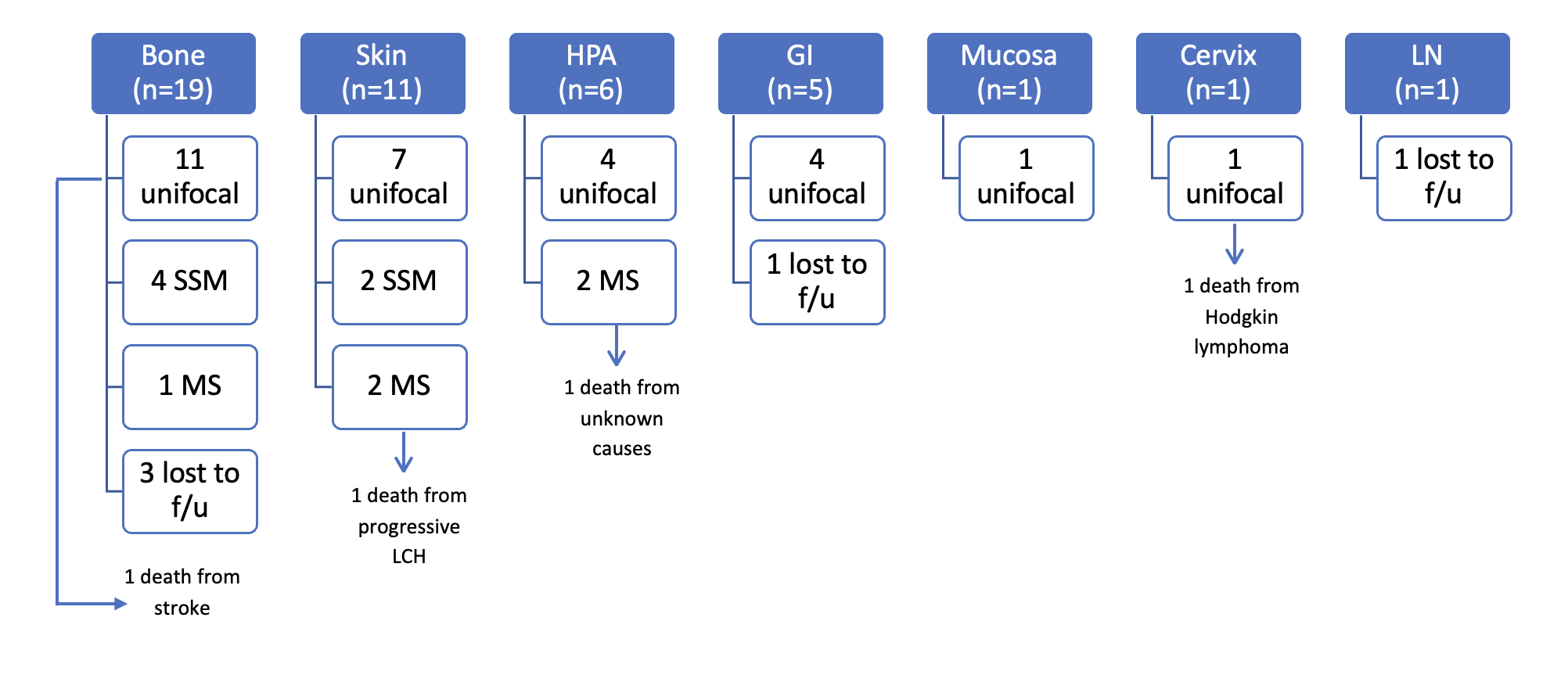
**

**Figure S7:** Non-pulmonary unifocal adult Langerhans cell histiocytosis: A) Progression-free survival (PFS) after first line therapy in years. B) Overall survival (OS) from time of diagnosis in years.

**
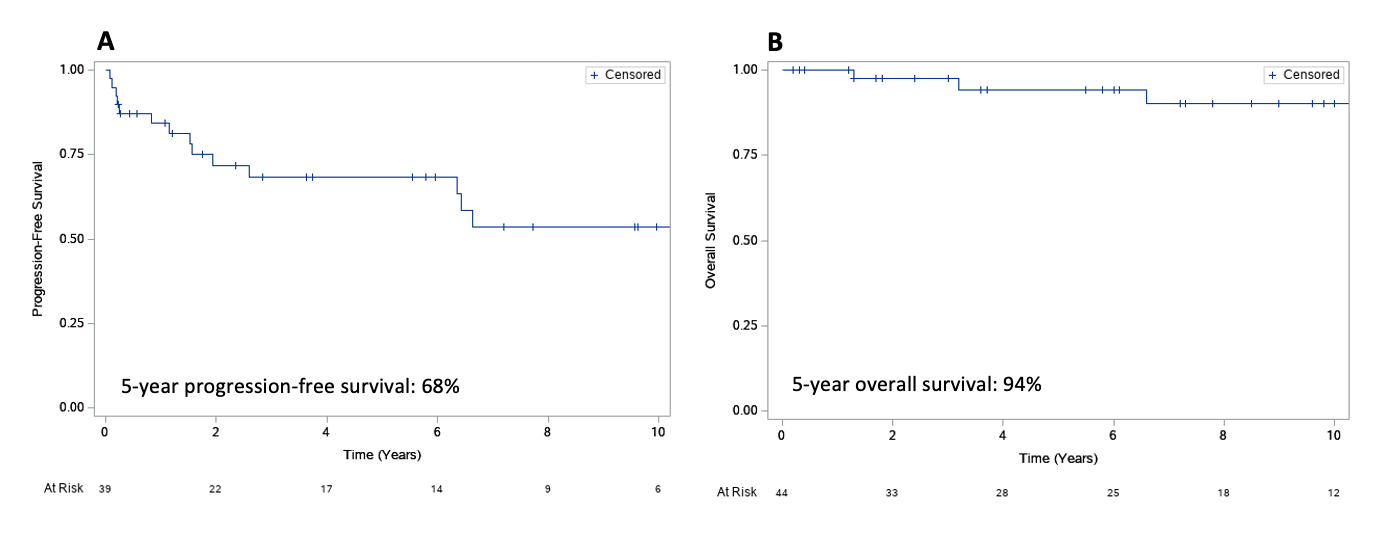
**
